# Supplementary material for: Evolutionary Conflict Leads to Innovation: Symmetry Breaking in a Spatial Model of RNA-Like Replicators
Source: Life (Basel). 2017 Nov 2;7(4):43. doi: 10.3390/life7040043 (PMC5745556; doi:10.3390/life7040043)
Supplement: Supplementary file 1 [file life-07-00043-s001.zip › Paper_Supp.pdf]

Supplementary Materials: Evolutionary Conflict Leads to Innovation: Symmetry Breaking in a Spatial Model of RNA-Like Replicators

Samuel H. A. von der Dunk*, Enrico Sandro Colizzi and Paulien Hogeweg *

1. Supplementary Figures

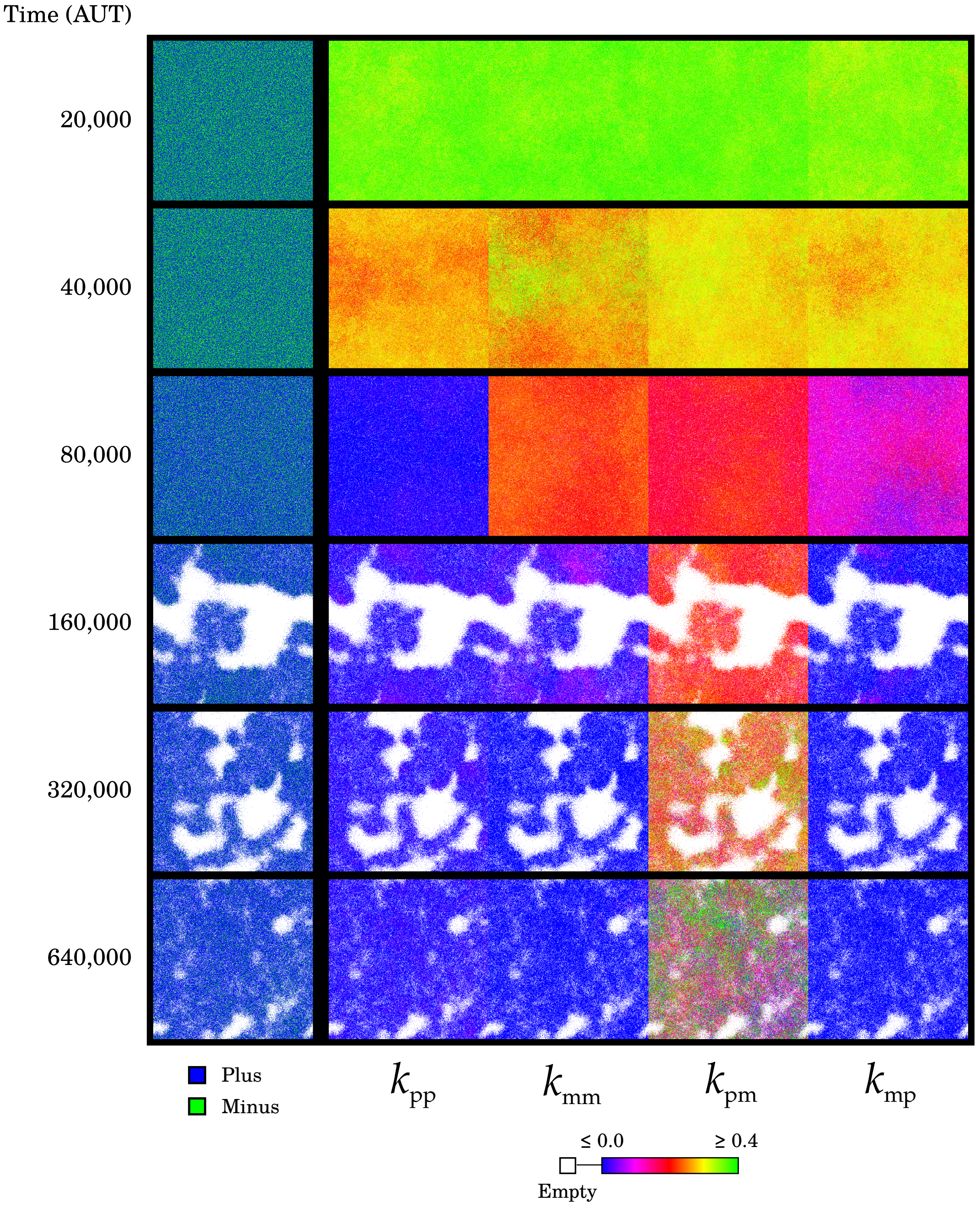

Figure S1. Evolutionary dynamics of the full model over time. For each site on the lattice, if it is a replicator, the strand type is shown (far left) and its value of k_{pp}, k_{mm}, k_{pm} and k_{mp} (from left to right). After a stage with only template asymmetry ($T = 80,000$), replicators become catalytically asymmetric as well.

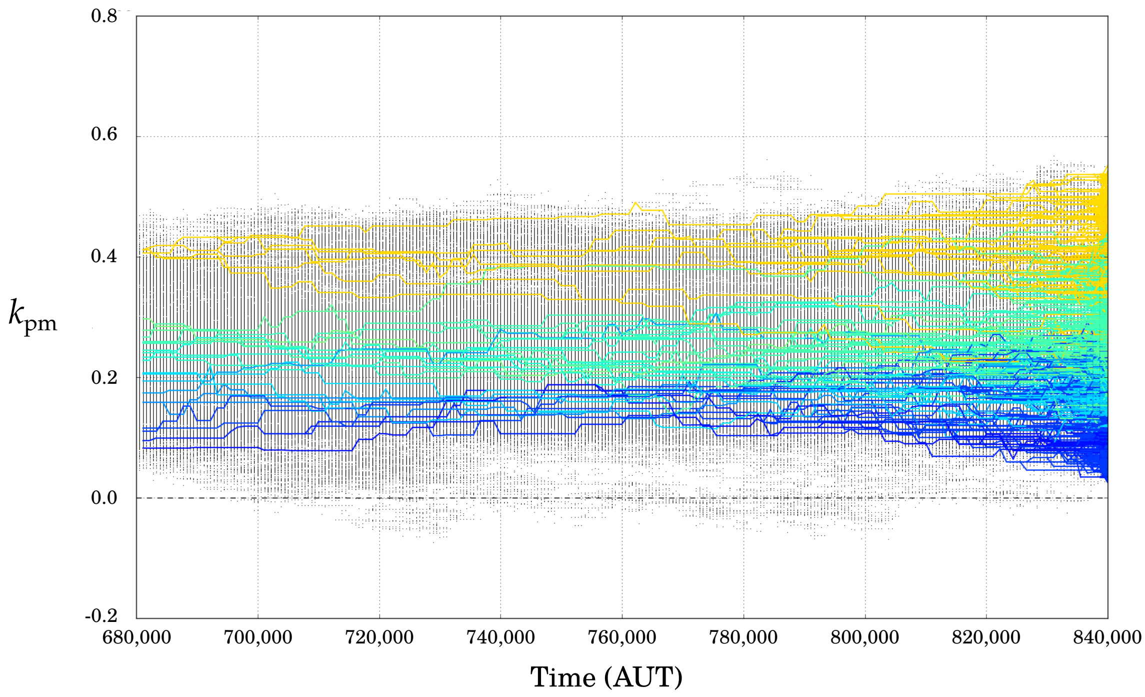

(a)

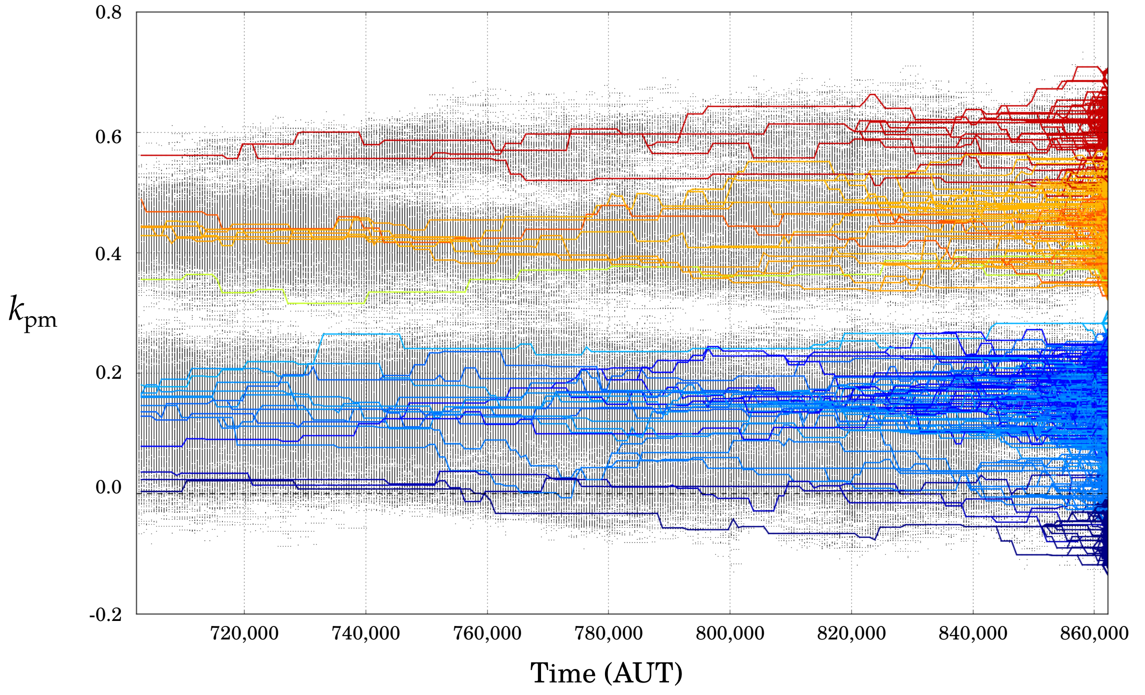

(b)

Figure S2. Ancestor traces of the full model for (**a**) $D = 50$ and (**b**) $D = 70$. For all replicators present at the end of the simulation the ancestors were traced back 160,000 AUT. Lineages are colored according to the value of k_{pm} of the first ancestor. The figure reveals the coexistence of several lineages with different values of k_{pm}.

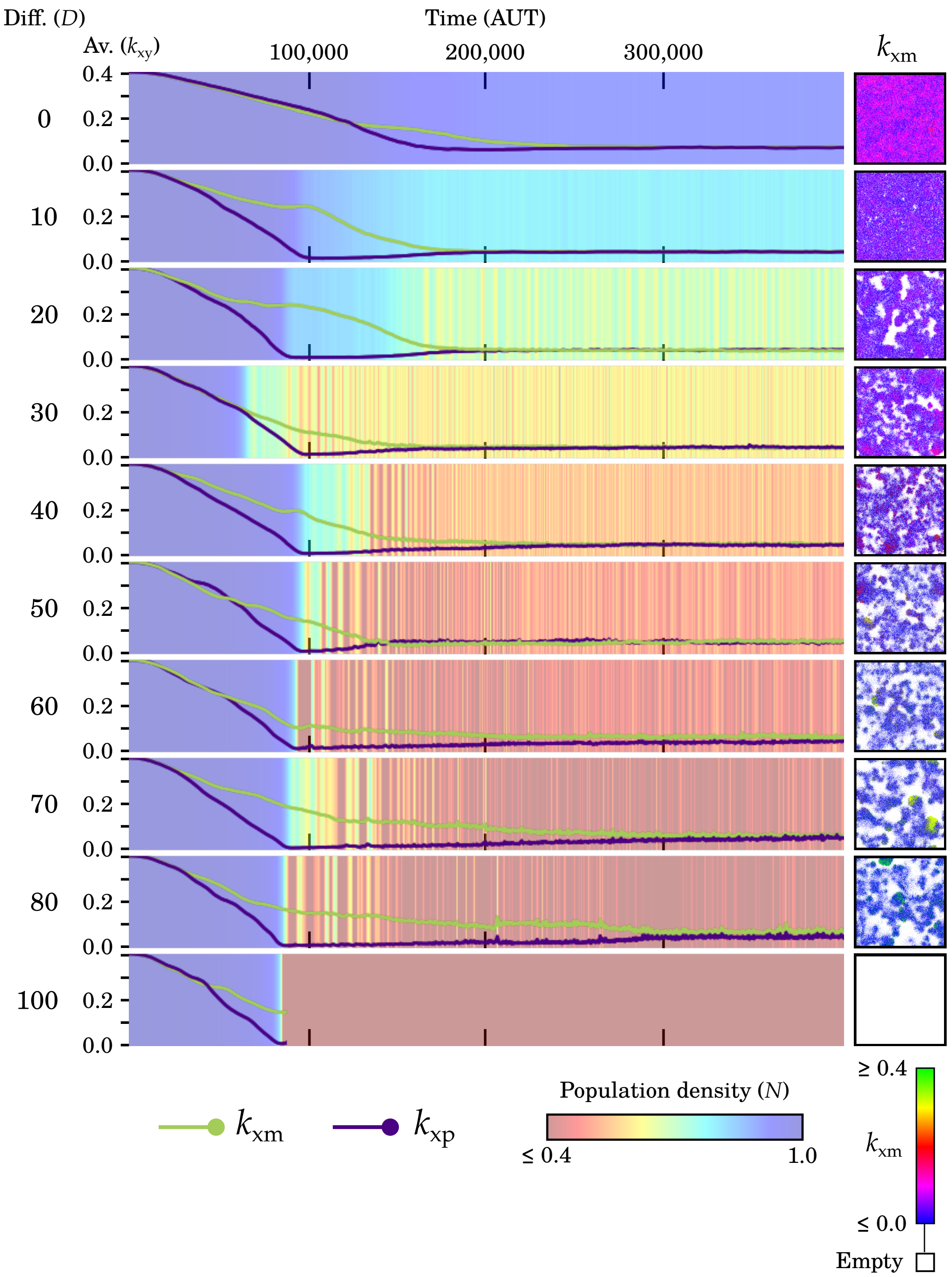

Figure S3. Evolutionary dynamics of the template model for different diffusion rates (see Figure 2 for clarification). The right panel shows a snapshot of the field at the end of the timeplot with replicators colored according to their value of k_{xm}. Template asymmetry suffices for survival at high diffusion, but the population density is low in these cases (cf. Figure 2).

2. Supplementary Videos

2.1. Video S1: Evolution at Low Diffusion

The evolution of k_{pm} is shown over time ($T = 0$ to $T = 166,667$) for $D = 10$.

2.2. Video S2: Evolution at High Diffusion

The evolution of $k_{\rm pm}$ is shown over time ($T = 0$ to $T = 166,667$) for $D = 70$. The video is slowed down 4 times compared to Video S1.

2.3. Video S3: Evolution of Two Competing Species

The evolution of the catalyst model competing with the basic model is shown. From left to right, for each site the following is shown: the strand type, the value of $k_{\rm px}$ in the catalyst model, the value of $k_{\rm mx}$ in the catalyst model and the value of $k_{\rm xx}$ in the basic model. The species form patches and a wave-pattern emerges.

2.4. Video S4: Competition at the Wave-Front

We let catalytically asymmetric replicators (blue) compete with symmetric replicators (green) for a stretch of empty space. Asymmetric replicators invade faster, outcompeting symmetric replicators at the wave-front.
